# The Evaluation and Management of Coronary Artery Disease in the Lung Transplant Patient

**DOI:** 10.3390/jcm12247644

**Published:** 2023-12-13

**Authors:** Gregory Serrao, Manish Vinayak, Johny Nicolas, Varsha Subramaniam, Ashton C. Lai, Daniel Laskey, Annapoorna Kini, Harish Seethamraju, Scott Scheinin

**Affiliations:** Icahn School of Medicine at Mount Sinai, New York, NY 10029, USA; manish.vinayak@mountsinai.org (M.V.); johny.nicolas@mountsinai.org (J.N.); varsha.subramaniam@mountsinai.org (V.S.); ashton.lai@mountsinai.org (A.C.L.); daniel.laskey@mountsinai.org (D.L.); annapoorna.kini@mountsinai.org (A.K.); harish.seethamraju@mountsinai.org (H.S.); scott.scheinin@mountsinai.org (S.S.)

**Keywords:** coronary artery disease, percutaneous coronary intervention, coronary artery bypass surgery, lung transplant evaluation

## Abstract

Lung transplantation can greatly improve quality of life and extend survival in those with end-stage lung disease. In order to derive the maximal benefit from such a procedure, patients must be carefully selected and be otherwise healthy enough to survive a high-risk surgery and sometimes prolonged immunosuppressive therapy following surgery. Patients therefore must be critically assessed prior to being listed for transplantation with close attention paid towards assessment of cardiovascular health and operative risk. One of the biggest dictators of this is coronary artery disease. In this review article, we discuss the assessment and management of coronary artery disease in the potential lung transplant candidate.

## 1. Introduction

Lung transplantation can truly be a life-saving procedure for the end-stage lung disease (ESLD) population. However, as with any organ allocation, comorbid conditions are important considerations prior to undertaking such a resource-intensive endeavor. Although there are many factors that go into the decision for lung transplantation, one of the more important considerations is concomitant coronary artery disease (CAD). Patient not only need to tolerate a complex transplantation procedure but they will also need to live long enough from a cardiovascular standpoint in order derived benefit from the transplantation.

Thus, coronary artery disease remains a relative contraindication to lung transplantation; however, with improvements in contemporary percutaneous and surgical revascularization strategies, the morbidity and mortality of underlying CAD in ESLD patients hopefully can now be successfully managed prior to, or even during, transplantation [1,2]. However, patients remain high-risk for recurrent coronary artery disease even after transplantation, as there is an accelerated atherosclerotic process seen in transplant recipients that is likely attributable to the immunosuppressive medications used [3]. Thus, surveillance, diagnosis, and treatment strategies need to be implemented even after lung transplantation.

Furthermore, there are significant interactions between CAD, end-stage lung disease, and lung transplantation that complicate a simplistic approach to clinical decision making. Therefore, given the high prevalence and significant morbidity and mortality of CAD in ESLD patients, careful attention to risk mitigation via pharmacological, procedural, and surgical approaches is important prior to, during and even after lung transplantation [4]. Given the complexity of management over such a long period of time, a multidisciplinary team is of the utmost importance. The involvement of a cardiologist is crucial in the lung transplantation process: diagnosis of CAD with the appropriate anatomical and/or functional stress test is fraught with nuisances: the proper management of CAD with the appropriate stent, antiplatelet therapy, anti-anginal therapy, and surgical revascularization approach are not trivial clinical considerations in the ESLD or transplanted population.

Here, in this review, we will discuss the impact of concomitant CAD in patients with end-stage lung disease, the diagnostic approach, medical therapy for primary or secondary prevention of CAD, the impact of percutaneous interventions of obstructive CAD, the impact of surgical interventions of obstructive CAD, and finally, post-lung transplantation management of CAD.

### 1.1. Prevalence of Coronary Artery Disease among Lung Transplant Candidates

**Figure 1:**
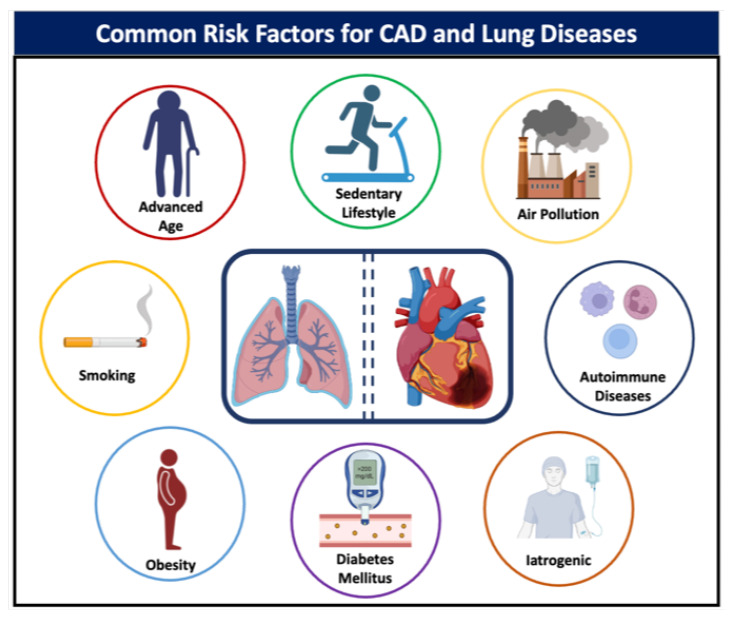
Common risk factors for coronary artery disease and lung disease.

The pathophysiological mechanisms that establish a connection between coronary artery disease (CAD) and advanced lung diseases remain inadequately understood. However, the relationship between these two medical conditions is principally attributed to overlapping risk factors, as illustrated in Figure 1. Despite an overall decrease in CAD in the general population, it is believed that its prevalence has increased among lung transplant candidates as more patients with advanced lung disease are now eligible for transplant compared to decades ago [5]. The prevalence of CAD among candidates for lung transplantation demonstrates considerable variation across different studies, ranging from 5% to 24% [1,6,7,8,9]. This substantial discrepancy can largely be attributed to the lack of a unified methodology for categorizing the severity of the disease. In most studies, CAD is defined based on coronary angiography, specifically as a luminal stenosis of either at least 50% or at least 70%. When the criteria are limited to patients exhibiting a 70% luminal narrowing in a major coronary artery, or a 50% narrowing in the left main coronary artery, the prevalence of CAD is approximately 10% [9]. The prevalence also appears to be influenced by the specific type of pulmonary disease; for instance, patients with fibrotic lung disease have twice the risk of developing CAD compared to those with non-fibrotic lung disease [10]. Moreover, the prevalence of CAD is significantly higher in patients with lung fibrosis than in those with emphysema (28.6% vs. 9.8%), even though the latter group demonstrates a higher prevalence of smoking. Risk stratification based on the presence of various comorbidities has revealed that individuals with at least three risk factors are more likely to exhibit obstructive CAD upon angiographic examination [7,9]. Specifically, diabetes mellitus and chest pain are associated with the highest positive predictive values, while hyperlipidemia and smoking are linked to the lowest [11]. One study, utilizing the Prospective Cardiovascular Münster (PROCAM) score to predict the risk of atherosclerotic disease in lung transplant candidates, found no correlation between various comorbidities and CAD. Most notably, this study revealed that nearly one-third of asymptomatic candidates over the age of 50 years were afflicted with undetected coronary artery disease [4]. Due to the inconsistencies in published reports and the absence of robust risk stratification tools, there is no uniform approach to the routine screening of occult CAD among patients undergoing pre-transplant evaluations. Nonetheless, most transplant centers currently screen lung transplant candidates who are above the age of 45 years.

### 1.2. Impact of Coronary Artery Disease on Lung Transplant Candidates

According to the most recent 2021 guidelines from the International Society for Heart and Lung Transplantation, having acute coronary syndrome within one month prior to transplant surgery, or possessing significant CAD that cannot be treated with revascularization, constitutes an important but not definitive disqualifier for undergoing a transplant [12]. The presence of CAD in lung transplant candidates introduces a complex layer of medical considerations, potentially increasing morbidity and mortality in the short and long term. As in any other non-cardiac surgery, CAD acts as a powerful negative prognosticator, imposing heightened risks for perioperative complications such as myocardial infarction, cardiac arrhythmias, and hemodynamic instability [13,14]. In the long term, immunosuppression accelerates the progression of atherosclerotic disease [15]. Despite that, data on the effects of preoperative CAD on mortality in lung transplant recipients are controversial. Several retrospective analyses conducted in the last half-decade indicate that lung transplant candidates with mild to moderate CAD, or those who have received revascularization procedures for CAD, may not necessarily experience increased perioperative complications or poorer survival outcomes compared to individuals without CAD [1,5,16,17]. In 210 lung transplant recipients, there were no mortality differences in the first 5 years after transplant between patients with mild-to-moderate CAD and patients without CAD [18]. A similar finding was reported in another study, including a cohort of 539 lung transplant candidates of whom one-third had moderate CAD [19]. Conversely, Jones et al. reported higher mortality rates in patients with CAD than in those without CAD following the transplant; however, the investigators used a broader definition of CAD, including those with a prior history of MI and revascularization [20]. To note, patients in these studies were meticulously selected and underwent more often a single-lung transplant. With respect to other post-transplant outcomes and complications, such as primary graft failure, prolonged hospital stay, myocardial infarction, and stroke, several studies have shown no differences between those with and without CAD, though the former group had higher rates of revascularization [2,21,22]. In one study including 539 lung transplant candidates, 33% had mild-to-moderate CAD and were more likely to undergo coronary revascularization following transplantation, as compared to those without CAD at baseline (PCI: 4.0% vs. 0.3%; CABG: 2.3% vs. 0.3%) [19]. Other cardiovascular events, not limited to coronary events but also occurring in other vascular beds, have been described. Few analyses report an increased incidence of non-fatal cardiovascular events, such as ischemic stroke and dysrhythmias, in lung transplant patients with CAD [8,16,23,24]. In a cohort of 280 lung transplant candidates, 48 patients with advanced CAD had an event rate of 12%, which consisted of 2% peripheral artery disease events, 4% coronary events, and 6% cerebrovascular events [16].

### 1.3. Medical Therapy for the Prevention or Treatment of CAD in Lung Transplant Candidates

**Figure 2:**
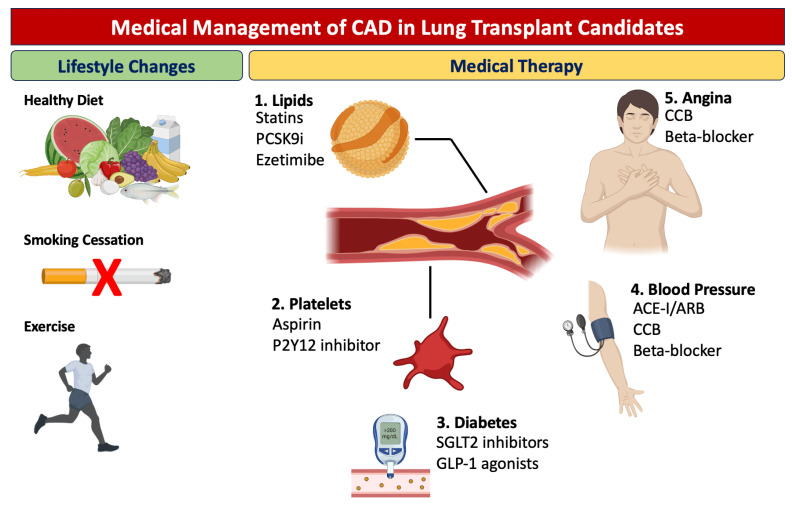
Medical management of coronary artery disease in lung transplant candidates.

Medical therapy aimed at treating and preventing CAD in lung transplant patients is of paramount importance, not only to improve perioperative cardiovascular risks but also to optimize long-term post-transplant survival (Figure 2). Lifestyle modifications, including smoking cessation, healthy diet, and exercise, can have a direct impact on cardiovascular risk in these patients. The cornerstone of medical management includes pharmacotherapeutic interventions such as antiplatelet agents (e.g., aspirin), lipid-lowering therapies, antihypertensive medications, and glucose-lowering agents in diabetic patients. These therapies aim to mitigate the underlying risk factors for CAD and have been shown to be effective in reducing the rates of myocardial infarction, stroke, and cardiovascular mortality. It is imperative that individualized medical regimens be tailored for each candidate, taking into account their specific cardiovascular and pulmonary pathology, and that continuous monitoring be instituted to assess treatment efficacy and adjust dosages as required. In patients with established CAD, low-dose aspirin is recommended for primary prevention [25]. The decision to initiate low-dose aspirin should be an individualized one, made in conjunction with a healthcare provider based on risk-benefit assessment. When aspirin is contraindicated, an adenosine diphosphate receptor (P2Y12) inhibitor may be used [26]. Statin treatment is the principal therapy focused on lowering cholesterol levels for reducing the risk of cardiovascular events in individuals with stable CAD, and it should be given at a dose that attains a targeted low-density lipoprotein cholesterol level [27,28]. When cholesterol levels are still above target, other agents such as ezetimibe and proprotein convertase subtilisin/kexin type 9 inhibitors may be added [29,30,31]. A recommended goal for systolic and diastolic blood pressure in individuals with CAD is below 130/80 mm Hg. The guidelines advise the preferential use of angiotensin-converting enzyme inhibitors or angiotensin receptor blockers for reducing the likelihood of cardiovascular incidents, with the prescription of β-blockers for patients with angina [32,33]. A tight control of sugar levels is an essential part in mitigating the cardiovascular risk; antidiabetic agents such sodium-glucose cotransporter 2 inhibitors (SGLT-2i) or glucagon-like peptide 1 receptor agonists have been shown to have a direct impact on the cardiovascular risk and should be preferred [34]. Lastly, antianginal medications, such as β-blockers and calcium channel blockers, are essential in symptomatic treatment. The effectiveness of various antianginal medications is generally modest and relatively comparable across different drug classes [35,36]. Consequently, the choice of medication is often determined by the presence of comorbid conditions and the likelihood of adverse effects. For individuals with left ventricular dysfunction or a history of myocardial infarction, β-blockers are particularly recommended. Newer agents, such as SGLT-2i and angiotensin receptor–neprilysin inhibitors, have also been shown to decrease mortality and hospitalization for heart failure and are currently recommended by guidelines for patients with left ventricular dysfunction [37].

### 1.4. CAD Screening in Lung Transplantation Candidates

Due to the scarcity of the donor lungs and concerns about the potential progression of underlying atherosclerosis with immunosuppressive therapies, guidelines have historically considered CAD as an absolute contraindication for the lung transplantation [38]. However, in light of advancements in procedural techniques, the 2014 update of the Pulmonary Transplantation Council of the International Society for Heart and Lung Transplantation guidelines considers CAD as an absolute contraindication only if there is uncorrected atherosclerosis accompanied by end-organ dysfunction and/or CAD that is not amenable to revascularization [39]. Subsequently, the 2021 update of these guidelines considers acute coronary syndrome or myocardial infarction within 30 days as the only absolute contraindication [12].

**Figure 3:**
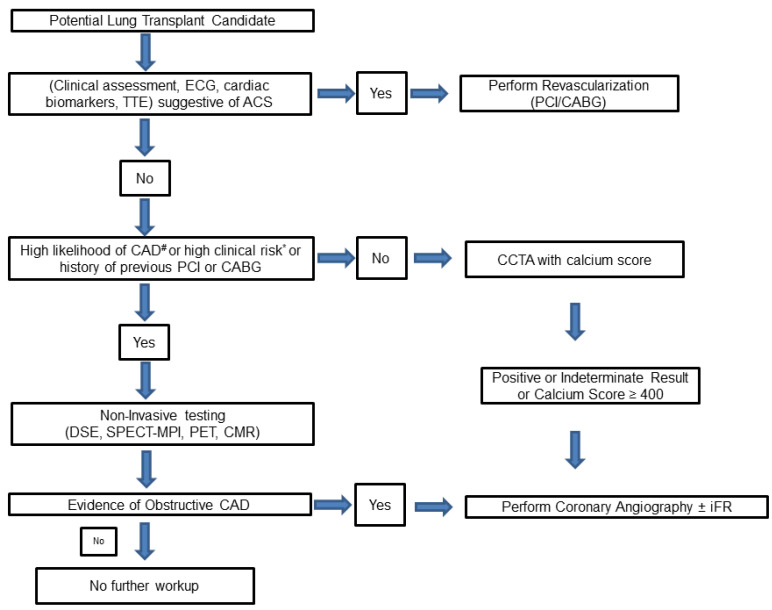
Proposed algorithm for protocolized assessment of coronary artery disease in lung transplant candidates. #: Two or more risk factors for cardiovascular disease (CVD): dyslipidemia, diabetes, hypertension, smoking, family history of CVD, prior established CVD. *: One or more clinical risk factor according to the Revised Cardiac Risk Index (RCRI): ischemic heart disease, cerebrovascular disease, history of congestive heart failure, serum creatinine level > 2 mg/dl, diabetes requiring insulin therapy. CABG: Coronary Artery Bypass Grafting, CCTA: Coronary Computed Tomography Angiography, CMR: Cardiac Magnetic Resonance, DSE: Dobutamine Stress Echocardiogram, ECG: Electrocardiogram, iFR: instantaneous wave-free pressure ratio, PCI: Percutaneous Coronary Intervention, PET: Positron Emission Tomography, SPECT-MPI: Single Positron Emission Tomography-Myocardial Perfusion Imaging, TTE: Trans-Thoracic Echocardiogram.

The evaluation prior to transplantation commences with an accurate history, a clinical examination, and an assessment of traditional cardiac risk factors, including age > 65 years, smoking, dyslipidemia, hypertension, diabetes, and a history of prior established cardiovascular disease. The presence of ≥2 cardiac risk factors correlates with a high likelihood of having underlying CAD [19]. Furthermore, a number of screening modalities (non-invasive and invasive) are available for the assessment of CAD risk, each with their own limitations (Figure 3).

#### 1.4.1. Exercise Testing

In terms of evaluating functional status, exercise testing has limited value due to poor functional capacity caused by underlying pulmonary disease, leading to an inability to achieve the target heart rate.

#### 1.4.2. Dobutamine Stress Echocardiogram (DSE)

Pharmacological stress testing with DSE is an accepted method for the preoperative assessment of cardiac risk in patients undergoing non-cardiac surgery. The utility of DSE in patients with severe lung disease has been evaluated in patients undergoing lung volume reduction surgery, where DSE exhibited limited positive predictive value (25%) but excellent negative predictive value (NPV) (100%) for perioperative and long-term cardiac events [40].

#### 1.4.3. Myocardial Perfusion Imaging (MPI)

Myocardial perfusion imaging with single positron emission tomography (SPECT) is another modality for diagnosing CAD in the general population. The sensitivity of MPI is as low as 43% with a reasonable specificity of 82% reported in a study on 349 patients undergoing lung transplant evaluation [41]. Furthermore, the role of pharmacological SPECT-MPI testing with vasodilators such as adenosine and dipyridamole is limited, due to possible receptor-mediated bronchoconstriction in patients with severe lung disease. Similar to DSE, dobutamine thallium MPI scan has limited specificity in lung transplant candidates with reported false-positive rates of up to 50% [42]. However, SPECT-MPI with regadenoson (a selective A2A receptor agonist) has been shown to be well tolerated in patients with end-stage lung disease with a negative predictive value (NPV) of 85% and a positive predictive value (PPV) of 75% [43]. Nonetheless, positron emission tomography (PET) for myocardial perfusion using rubidium-82 has a high sensitivity and specificity of 90% and 88%, respectively, in detecting CAD in the general population and is recommended as one of the imaging modalities for patients undergoing solid organ transplantation, including lung transplantation [44].

#### 1.4.4. Stress Cardiac Magnetic Resonance (CMR)

Stress CMR imaging along with late gadolinium enhancement has been shown to predict myocardial ischemia, along with assessment of myocardial viability and cardiac function. A meta-analysis involving 2841 patients with CAD revealed that stress CMR exhibits a pooled sensitivity of 89% and a specificity of 76% in predicting CAD with ≥50% stenosis on coronary angiography [45].

#### 1.4.5. Coronary Computed Tomography Angiography (CCTA)

Coronary computed tomography angiography (CCTA) is validated as a potential alternative to coronary angiography for diagnosing and grading the severity of CAD. A meta-analysis of studies evaluating the ability of CCTA and coronary artery calcium score (CACS) to predict perioperative cardiovascular events in non-cardiac surgery has demonstrated a threefold and eightfold increased risk of perioperative major adverse cardiovascular events (MACE) for individuals with single-vessel and multivessel obstructive CAD on CCTA, respectively, as compared to those with no CAD. Furthermore, a rising CACS showed a correlation with a progressively elevated trend in perioperative MACE (CACS ≥ 100, odds ratio (OR) of 5.2; ≥1000, OR of 10.4; both with a *p*-value < 0.01) [46]. While one study assessed the usefulness of CACS assessment on high-resolution computed tomography (HRCT) for patients with idiopathic pulmonary fibrosis, there is a paucity of data regarding the predictive ability of CCTA and CACS with respect to perioperative outcomes in patients undergoing lung transplantation [47].

#### 1.4.6. Coronary Angiography

Invasive coronary angiography is the gold standard for CAD detection but is associated with procedural risks, including radiation and contrast exposure. Newer assessment strategies such as physiology assessment with fractional flow reserve (FFR), plaque characterization with intra-vascular ultrasound (IVUS), and optical coherence tomography (OCT) improve diagnostic accuracy. Moreover, newer techniques such as the instantaneous wave-free pressure ratio (iFR) and the Resting flow ratio (RFR), which do not require adenosine, offer an additional advantage in assessing the significance of intermediate stenosis in lung transplant candidates, as adenosine has the potential to induce bronchospasm [48].

The choice between the invasive versus non-invasive screening modalities remains an elusive one. The 2022 ESC guidelines for cardiovascular assessment in non-cardiac surgery clearly categorize invasive coronary angiography as a Class III recommendation for patients undergoing low- or intermediate-risk non-cardiac surgery [49]. However, in the case of lung transplant, which is categorized as high surgical risk (30-day risk of cardiovascular death, myocardial infarction (MI), and stroke > 5%), there is a lack of clear guideline recommendations regarding the use of invasive coronary angiography as part of preoperative evaluation in this specific population. Due to the lack of guideline recommendations, as well as the questionable sensitivity and specificity of non-invasive testing, many lung transplant centers continue to favor coronary angiography as the screening investigation of choice, particularly for individuals over the age of 40. Figure 1 represents a possible approach to CAD screening in lung transplantation candidates. This represents our personal standpoint on the evaluation of coronary artery disease, with an emphasis on the importance of future guideline recommendations.

### 1.5. Indications for Revascularization

The quandary of whether to undergo coronary angiography extends to the question of whether revascularization should be considered for asymptomatic patients. The occurrence of CAD in patients undergoing non-cardiac surgeries places them at higher risk of ischemic events during the perioperative period. However, there are conflicting data regarding the benefit offered by prophylactic PCI before non-cardiac surgery in reducing perioperative ischemic events [50]. Routine revascularization prior to non-cardiac surgery in order to reduce perioperative cardiovascular events is currently not recommended under the 2014 ACC/AHA guidelines and the 2022 ESC guidelines (unless undergoing carotid endarterectomy) [49,50,51]. Therefore, the recommendations for cardiac revascularization before non-cardiac surgeries remain the same as they would be if surgery was not a factor [51].

Various studies have reported similar rates of survival with no differences in perioperative morbidity between patients with obstructive CAD requiring revascularization, either by PCI or previous or concomitant CABG, compared to those with no underlying CAD [5,52]. Similarly, for patients with moderate CAD (angiographic epicardial stenosis > 20% but <70%), Zanotti et al. and Choong et al. have reported that there is no significant increase in perioperative morbidity or mortality in patients with moderate CAD who do not undergo preoperative revascularization before lung transplantation compared to patients with no underlying CAD. However, among moderate-CAD patients, 6% to 18% developed symptomatic artery disease requiring post-transplant revascularization by either CABG or PCI, highlighting the importance of initiating statins and aspirin to prevent cardiovascular events in patients with underlying CAD [18,19].

### 1.6. Percutaneous Coronary Intervention (PCI)

The decision for surgical versus percutaneous revascularization should be a discussion between the interventional cardiologist, the cardiothoracic surgeon, and the lung transplantation team. For example, patients with multivessel disease would likely derive more benefit with surgical revascularization at the time of lung transplantation surgery than with percutaneous intervention prior to surgery. Additionally, stenting of complex lesions (i.e., bifurcation lesions) should be avoided as these lesions tend to have poorer procedural success and worse early and late outcomes. In contrast, simple lesions can be addressed prior to lung transplantation via a percutaneous approach with excellent results. Stent type, antiplatelet therapy, and approach to stenting should all be taken into account when the decision for a percutaneous approach is made.

#### 1.6.1. Stent Choice

The choice of stent (bare metal stent versus drug-eluting stent) used to be much more of a complex decision as the stent material would dictate the duration of postprocedural antiplatelet regimen, with significant differences in the rates of in-stent thrombosis and in-stent restenosis. However, the newer generation drug-eluting stents (DESs) have a similar, if not reduced, risk of in-stent thrombosis as compared to bare metal stents; furthermore, there is an added benefit of longer stent patency with the newer generation DESs when compared to BMSs [53]. Thus, BMSs are rarely used in the modern-day approach to coronary revascularization.

Under the previous 2016 AHA/ACC guidelines, de-escalation from dual-antiplatelet therapy (DAPT) can be considered after 1 month for BMSs with stable coronary artery disease. This was in comparison to the 3–6 months DAPT regimen for DESs in stable coronary artery disease. However, under the current AHA/ACC guidelines, de-escalation from dual-antiplatelet therapy (DAPT) to P2Y12 inhibitor monotherapy can be considered in select patients after 1–3 months of the DAPT regimen for DESs in stable coronary artery disease [54,55]. This recommendation is based on data suggesting that a shorter duration of DAPT may be sufficiently protective against in-stent thrombosis of newer generation DESs in patients with a high bleeding risk [56,57]. This 1-month duration of DAPT has also been reflected in the 2018 European guidelines [58]. Thus, this shortened duration of DAPT for the newer generation of DESs may provide the most optimal balance between the urgency of lung transplantation, minimizing the risks of perioperative bleeding and early in-stent thrombosis, while providing longer term stent patency.

#### 1.6.2. DAPT Choice

As discussed above, the duration of post-procedural dual antiplatelet therapy (DAPT) significantly impacts the timing of lung transplantation listing. Thus, prior to percutaneous revascularization, the need for DAPT in the post-procedural immediacy needs to be taken into account. Interrupted antiplatelet therapy for emergent lung transplantation can lead to in-stent thrombosis. However, continuation of antiplatelet therapy during a high-risk surgery, such as lung transplantation, can lead to an unacceptable risk of perioperative bleeding. Therefore, close collaboration between the transplantation pulmonologist, the non-invasive cardiologist, and the invasive cardiologist is essential in the approach to coronary artery disease prior to lung transplantation.

Antiplatelet therapy is generally required in the subsequent weeks to months after stent placement. The clinical context in which the stent was placed also affects the likelihood of in-stent thrombosis during high-risk surgery. Those who were revascularized urgently due to acute coronary syndrome are at greatest risk of in-stent thrombosis with interruption of DAPT. Thus, antiplatelet therapy is an especially important consideration in cadaveric lung transplantation. The uncertainty of lung transplantation timing creates a complex clinical conundrum. As with most clinical scenarios involving emergent procedures and antiplatelet therapy, there is a difficult balance between increased risk of perioperative bleeding, risk of in-stent thrombosis, and the life-saving transplantation. In the setting of recent coronary stenting, there is little time to transition to a short-acting formulation such as cangrelor for perioperative bridging if a cadaveric lung becomes available for transplantation. In one clinical scenario, an ESLD patient could present with acute coronary syndrome requiring emergent stenting and ensuing months of long, uninterrupted DAPT; however, that same patient may now no longer be a candidate for life-saving lung transplantation given the prohibitive surgical bleeding risk on DAPT.

With regard to longer term antiplatelet management after stenting, certain P2Y12 inhibitors may be more detrimental in the end-stage lung disease population. For example, patient dyspnea attributed to increased endogenous levels of adenosine is a known side effect of ticagrelor [59]. Though there is no clear causal evidence that ticagrelor itself has any detrimental effect on pulmonary function at rest or during exercise, ticagrelor-induced dyspnea may be prohibitive side effect in an already at-risk population. Furthermore, ticagrelor-induced dyspnea may also act as a confounder to worsening pulmonary function in end-stage lung disease [60]. In contrast, prasugrel is fairly well tolerated and is not associated with dyspnea or any lung injury [61,62,63]. In a similar vein, clopidogrel is also well tolerated by most patients. Despite being the most common P2Y12 inhibitor prescribed, there are only a handful of case reports of clopidogrel-associated interstitial lung disease [64,65]. Thus, it would seem more prudent to try to utilize clopidogrel and prasugrel in the ESLD population if patients are responders without absolute contraindications to the agents.

#### 1.6.3. Stent Optimization

In addition to the type of stent and antiplatelet regimen, there are also important considerations with regard to stent optimization in order to further reduce the risk of in-stent thrombosis in the immediacy of stent placement and/or longer term in-stent restenosis.

The advent of intravascular imaging with intravascular ultrasound (IVUS) and optical coherence tomography (OCT) has allowed an interventional cardiologist to almost perfectly size the diameter and length of a stent. This is a crucial development as stent undersizing is a consistent predictor of stent thrombosis and/or restenosis. Furthermore, coronary calcification is a major cause of stent undersizing. Compared to using coronary angiography alone, intravascular imaging can not only help with sizing but also quantify plaque burden and plaque characterization, such as calcification [66]. Finally, intravascular imaging is also useful in more ambiguous cases where the lesion severity by coronary angiography is borderline [66]. Thus, intravascular imaging has now emerged as a pivotal technique in an interventionalist’s toolbox.

For lesions that are amenable to percutaneous intervention, imaging guidance with IVUS or OCT can be useful for stent optimization. The use of intravascular imaging whether by IVUS or OCT has been associated with improved outcomes, not only at vessel level optimization but also in major cardiovascular outcomes. Intravascular imaging-guided DES implantation has been associated with lower rates of target lesion revascularization, target vessel revascularization, and stent thrombosis [67]. Furthermore, intravascular imaging guidance using either IVUS or OCT was associated with a significant reduction in major adverse cardiovascular events and cardiovascular death [68,69]. More recent promising data also suggest that OCT guided revascularization can provide further clinical benefit for complex lesions (i.e., bifurcation lesions) and result in a larger minimum stent area compared to angiography alone [70,71]. Given the resource-intensive nature of lung transplantation, it only seems reasonable to utilize the most appropriate tools to optimize ESLD patients for the best possible short-term and long-term revascularization outcomes. Furthermore, intravascular imaging is becoming routine in many cases given the plethora of supporting data; its use under particular situations has now been incorporated into the 2023 guidelines [54,55,72,73]. In all reality, intravascular imaging may soon become the new standard for percutaneous coronary interventions given the plethora of supporting data [72].

With improvement in vessel revascularization and stent thrombosis using intravascular imaging guidance, there are ongoing trials to address whether image-guided PCI would permit the reduction in DAPT duration [74]. If findings of these trials are favorable, the results would have important implications in the ESLD patient population. A shortened duration of DAPT with intravascular imaging may allow more flexibility in lung transplantation listing. If a safe, rapid de-escalation of DAPT can be permitted, then the risk of perioperative bleeding for lung transplantation will concordantly be reduced at an earlier time point after percutaneous intervention.

Despite the developments in percutaneous revascularization, not all lesions are amenable to such approach. There remains a large role for surgical revascularization for risk mitigation and survival benefit, especially in complex coronary artery disease.

### 1.7. Surgical Revascularization Coronary Artery Bypass Grafting (CABG)

Those with complex coronary artery disease (i.e., bifurcation lesions, multivessel disease), patients may be better served with surgical revascularization. Furthermore, a surgical revascularization, as compared to a percutaneous approach, would abrogate the necessity of prolonged DAPT and the ensuing deference of lung transplantation listing. Once the decision is made for a surgical revascularization strategy, surgical revascularization can occur prior to or at the time of transplantation.

#### 1.7.1. Approach to Surgical Revascularization

Although revascularization can be done safely a few days prior to transplantation, a review of literature suggests that most centers elect for surgical revascularization during transplantation [22,75]. In these cases, surgical revascularization was performed just prior to lung transplantation.

Recipients of concurrent surgical revascularization and lung transplantation appear to have similar morbidity and mortality to recipients of lung transplantation alone [76,77]. Surgical consideration for concomitant revascularization included longer on-pump duration time, longer operative time, and incision into the pericardium. The recovery after concomitant coronary revascularization and lung transplantation has also been demonstrated to be longer, with longer postoperative length of stay, longer time in the intensive care unit, and more days on ventilator support [78]. However, the overall survival appears to be similar as compared to those patients who underwent revascularization with PCI prior to transplantation [22].

Both revascularization and lung transplantation can be accomplished off cardiopulmonary bypass [22,79]. If it can be performed safely, off-pump surgery for lung transplantation has been demonstrated to reduce the duration of ventilation, ICU stay, and hospital length of stay when compared to propensity-matched on-pump surgery [78]. However, some studies suggest that off-pump coronary revascularization has been associated with increased rates of incomplete revascularization compared to on-pump CABG [80,81]. Thus, the use of cardiopulmonary bypass depends on multiple clinical factors and varies among institutions depending on surgical expertise [78]. While cardiopulmonary bypass unloads the heart and improves hemodynamic stability, the ensuing possible coagulopathy, hemolysis, and activation of systemic inflammatory response may complicate the postoperative course. Interestingly, there are some data suggesting that intraoperative extracorporeal membrane oxygenation (ECMO) may decrease pulmonary, renal complications, and confer a survival benefit as compared to cardiopulmonary bypass [82,83,84,85]. In comparative studies, there was a higher anticoagulation, a higher need for transfusions, and higher in-hospital mortality with cardiopulmonary bypass as compared to ECMO [86]. Depending on local surgical expertise and multiple patient factors, ECMO can be used in particular situations and not routinely in place of cardiopulmonary bypass.

#### 1.7.2. Arterial Versus Venous Grafts

The current 1-, 5-, and 10-year survival after lung transplantation remains at 80%, 54%, and 32%, respectively, while the benefits of arterial revascularization over venous revascularization tend to be realized after many years [87,88,89]. The 10-year patency rates of saphenous vein grafts are of the order of 50–60% while the 10-year patency rates of internal mammary artery are upwards of 90% [90]. Given the expected survivability after lung transplantation, the long-term benefits of arterial grafts may not be completely realized after lung transplantation [91]. Thus, vein grafts may be sufficient to provide an upfront high-volume conduit while maintaining internal mammillary arteries for optimal healing after sternotomy. The extent and complexity of coronary artery disease should be taken into account when considering the usage of arterial grafts and vein grafts. This clinical decision should involve a multidisciplinary discussion between the cardiothoracic surgeon, cardiologist, and lung transplantation pulmonologist.

The management of CAD does not end after revascularization and lung transplantation. After revascularization, either percutaneously or surgically, lung transplantation patients still require significant medical therapy in order to reduce the recurrence of obstructive coronary artery disease. The management of DAPT after PCI has been described above; however, many of the other pharmacological therapies for CAD risk modification can interact with the immunosuppressive agents that are required post transplantation.

### 1.8. Immunosuppression and CAD in Lung Transplant Recipients 

To ensure the longevity and functionality of the transplanted organ, immunosuppressive medications remain the cornerstone of graft rejection prevention. Yet, these agents, however indispensable, come with an array of side effects. One particularly concerning sequela of prolonged immunosuppressive therapy is its association with the development and progression of coronary artery disease (CAD).

#### 1.8.1. Calcineurin Inhibitors (Cyclosporine and Tacrolimus)

Within the post-transplant community, CNIs have been linked to the development of several cardiovascular homeostatic dysregulations that put patients at elevated risk for development or progression of CAD. One of the most notable is the development of hypertension (HTN). For any transplant recipient, persistent HTN poses a significant risk for adverse outcomes including reduced allograft longevity, renal dysfunction, cardiac hypertrophy, and vascular dysregulation. Each of these complications individually, and even more so in combination, can contribute to CAD [92]. In lung transplant recipients, studies have shown that a majority of CNI-treated recipients without pre-existing cardiovascular risk factors develop post-transplant HTN [93]. This association is consistent for patients with other solid organ transplantation on CNI maintenance therapy [94].

Beyond HTN, CNIs, especially cyclosporine, have been recognized as contributors to post-transplant dyslipidemia [95]. Alterations in lipid metabolism are prevalent in all solid organ transplant recipient populations, with lung transplantation having a notable prevalence of approximately 40% [96]. Changes in lipid profiles with cyclosporine includes elevations in total cholesterol, LDL-C, non-HDL-C, triglycerides, apolipoprotein B, and apoO-III [95,97]. Additionally, cyclosporine has been shown to increase triglyceride levels by hindering their breakdown through the activity of lipoprotein lipase [98].

While CNI-induced lipid dysregulation is significant, the development of diabetes mellitus further heightens the risk of CAD, adding another dimension of cardiovascular concern for transplant recipients. Post-transplant diabetes mellitus (PTDM) is a common and potentially serious complication after solid organ transplantation. The prevalence of PTDM in lung transplant recipients varies across studies but is generally estimated to be around 10–40% within the first year [99]. Calcineurin is expressed in pancreatic β-cells. CNIs induce direct cell toxicity by targeting cAMP response element binding protein (CREB) and the NFAT family of transcription factors. CREB promotes expression of insulin signaling genes, such as glucagon-like peptide (GLP-1), and mediates β-cell survival [100]. CNIs prevent the phosphorylation of CREB and subsequent activation which leads to decreased insulin production and cellular apoptosis [101].

#### 1.8.2. Anti-Proliferative Agents (Mycophenolate Mofetil or Sodium and Azathioprine) 

Azathioprine (AZA) and mycophenolate mofetil (MMF) have the lowest cardiac toxicities of all maintenance immunosuppressive agents. Limited data exist for head-to-head comparison of AZA and MMF and development or progression of CAD. A study looking into mortality for heart transplant patients randomized to receive AZA or MMF found that patients treated with MMF had fewer fatal cardiovascular events and less autopsy-proven atherosclerosis [102]. AZA and MMF do not have significant effects on HTN, lipid dysregulation, or glucose metabolism [92,103].

#### 1.8.3. mTOR Inhibitors (Sirolimus and Everolimus) 

The mammalian target of rapamycin (mTOR) inhibitors are typically considered an adjunct or alternative therapy. In lung transplant recipients, mTOR inhibitors are often used as alternatives to CNIs or other antiproliferative agents, typically in cases of CNI-induced nephrotoxicity, bronchiolitis obliterans syndrome, or post-transplant lymphoproliferative disorder [104,105]. mTOR inhibitors have been shown to reduce vascular smooth muscle proliferation, intimal hyperplasia, and adhesion of endothelial cells secondary to pro-inflammatory signaling (IL-6, VEGF, TNFα) [106]. Endothelial injury and intimal hyperplasia have been implicated as causes of post-transplant-related CAD [107]. In heart transplant recipients, higher levels of TNF-α were associated with the development of CAD and cardiac allograft vasculopathy [108]. As such, mTOR inhibitors have been playing an increasingly important role in the prevention of transplant-associated CAD development. A randomized controlled trial comparing sirolimus with AZA in combination with cyclosporine and steroids reported a lower incidence of rejection and less luminal narrowing in patients in the mTOR treatment wing [109]. mTOR inhibitors have also repeatedly shown favorable HTN profiles compared to CNIs and therefore have been used for lung transplant populations with post-transplant HTN and for severe pulmonary arterial hypertension (PAH) [106,110]. Sirolimus and everolimus also appear to increase nitric oxide production, preventing endothelial dysfunction and hyperplasia [111].

#### 1.8.4. Corticosteroids 

Hyperlipidemia and diabetes can significantly elevate risk of coronary disease by promoting atherosclerosis and inducing chronic inflammation. Cumulative dosing of steroids appears to be the most significant risk factor for the development of hyperlipidemia and post-transplant diabetes in solid organ transplant recipients. Steroids can influence cholesterol levels through several mechanisms of action such as insulin resistance, increased VLDL secretion, decreased LDL receptor activity, increased cholesterol synthesis, and inhibition of lipoprotein lipase [112]. Steroids predispose patients to significant weight gain with long-term use. This leads to insulin resistance, which triggers hepatic secretion of VLDL. Steroids also downregulate the activity of hepatic LDL receptors, limiting the clearance of LDL cholesterol from the bloodstream. Moreover, they upregulate the activity of lipogenic enzymes such as acetyl-coenzyme A carboxylase and free fatty acid synthase, accelerating cholesterol synthesis. The activity of HMG-CoA reductase, a key enzyme in cholesterol synthesis, is also upregulated. The inhibition of lipoprotein and triglyceride breakdown by lipoprotein lipase contributes to higher circulating lipid levels. These mechanisms collectively contribute to the dyslipidemia seen in post-transplant recipients.

In a similar fashion, steroids increase peripheral insulin resistance, limit pancreatic insulin secretion, and inhibit hepatic glucose production [113]. These derangements lead to hyperglycemia and eventually diabetes. Studies have even shown an association between pulse-dose steroids for acute rejection and post-transplant diabetes [114]. Post-transplant diabetes is recognized as a cardiac risk equivalent, and significantly elevates risk of CAD. The co-occurrence with other risk factors such as hyperlipidemia can further exacerbate cardiovascular risk.

#### 1.8.5. Statins

The post-transplant population experiences an elevated overall cardiac risk, in part due to the required immunosuppressive medication aimed at preventing graft rejection. As a result, stains often find a place in the post-transplant regimen. By inhibiting HMG-CoA reductase, statins help lower circulating cholesterol levels. Beyond their favorable impact on lipid metabolism, statins have demonstrated potent inhibitory effects on pro-inflammatory cytokines and are postulated to enhance endothelial function against oxidative stress [115]. Notably, the use of statins has been associated with a reduced risk of primary graft dysfunction (PGD), a syndrome of acute lung injury occurring in the early post-transplantation stages and a leading cause of mortality within the first year [116]. Moreover, evidence suggests that statins’ antifibrotic effects might be beneficial in averting or managing bronchiolitis obliterans syndrome (BOS), a form of chronic allograft rejection that results in diffuse inflammation and scarring [117]. Given the substantial benefits that statins offer to transplant recipients, explorations are underway regarding the merit of preoperative statin administration as a protective factor against long-term graft dysfunction [118]. However, the post-transplant administration of statins requires attention due to the potential for significant drug-drug interactions.

In post-transplant recipients, the concurrent use of CNIs has been associated with increased incidences of statin-induced muscle injury, liver injury, and rhabdomyolysis [119,120]. Several pathways of interaction between CNIs and statins have been hypothesized, including the inhibition of CYP3A4 and other transporters, which potentially leads to reduced hepatic uptake and increased statin exposure in smooth muscles [121]. Cyclosporine, for instance, can inhibit CYP3A4 activity, thereby reducing the metabolism and elevating the levels of certain statins such as atorvastatin, simvastatin, and lovastatin. However, elevated circulating levels have also been observed with non-CYP3A4-dependent statins such as pravastatin and fluvastatin, indicating a potential secondary process [120]. It is hypothesized that the inhibition of hepatic transporter proteins such as PGP and OATP1B1 could lead to decreased hepatic uptake and increased plasma levels of statins. The 1B1 subtype of OATP serves as the primary distribution site for statins and targets their lipid-lowering actions [122]. This transporter facilitates statin uptake into hepatic cells, preventing their circulation in the bloodstream and thereby minimizing muscle toxicity risks. Pharmacokinetic studies have demonstrated a longer half and higher area under the curve (AUC) for statins in transplant recipients on CNIs compared to their non-transplant counterparts [122]. Intriguingly, small-scale in vitro and in vivo studies have also noted increased rates of statin-induced myopathy with cyclosporine compared to tacrolimus, attributing a significant decrease in hepatic and intestinal PGP, OATP1B1, and CYP3A4 activity due to cyclosporine, while a limited effect was observed with tacrolimus [122,123]. Current American Heart Association (AHA) recommendations advise against combining CNIs with lovastatin, simvastatin, and pitavastatin. Additionally, it is recommended that dosages of atorvastatin greater than 10 mg/day, when co-administered with CNI, should be routinely monitored for CK levels and signs and symptoms of muscle toxicity [124]. Given the findings regarding interaction with tacrolimus, it suggests that tacrolimus could be the safer CNI option and may not require dose adjustments. An alternative recommendation might be to use statins that are not dependent on CYP3A-mediated metabolism, such as fluvastatin, pravastatin, and rosuvastatin [95].

## 2. Conclusions

Recognition and treatment or coronary artery disease in the lung transplant recipient is critical to successful outcomes. Careful attention to prevention, assessment, treatment, and ongoing monitoring post transplantation can significantly improve outcomes in this patient population.

## Data Availability

Data are contained within the article.
